# Dynasore inhibition on productive infection of HIV-1 in commonly used cell lines is independent of transferrin endocytosis

**DOI:** 10.19185/matters.201805000001

**Published:** 2018-05-30

**Authors:** Hanna Song, Michael DeSantis, Chunjuan Tian, Wei Cheng

**Affiliations:** 428 Church Street, University of Michigan; Pharmaceutical Sciences, Biological Chemistry, University of Michigan

**Keywords:** Endocytosis, Dynasore, HIV, Biochemistry, Biophysics, Cell Biology

## Abstract

The route of HIV-1 entry for productive infection in CD_4+_ host cells is a fundamental question for the molecular understanding of HIV-1 infection and transmission. Although direct fusion has long been thought to be the mode of entry, recent studies have suggested that productive entry of HIV-1 may actually occur through dynamin-dependent endocytosis. In several of these studies, dynasore, a noncompetitive inhibitor of the GTPase activity of dynamin, has been used to support this conclusion. Here we show that dynasore does produce inhibitory effects on the productive infection of HIV-1 in several commonly used cell lines. This effect is present regardless of the methods used to facilitate the infection of HIV-1. However, transferrin uptake remains fully functional in these cell lines upon dynasore treatment. Therefore, the inhibition on HIV-1 infection by dynasore in these cell lines is due to an effect that is independent of transferrin endocytosis. The use of dynasore in probing the role of endocytosis in HIV-1 infection should be corroborated by other methods.

## Introduction

Dynamin is a large GTPase whose membrane fission activity is required for clathrin-mediated endocytosis and also some clathrin-independent endocytic processes [1] [2]. Dynasore, originally discovered as a noncompetitive inhibitor of the GTPase of dynamin [3], has been widely used as a cell-permeable and fast-acting inhibitor to study the function of dynamin in various cellular events. Recently in several studies [4] [5] [6] [7] [8], dynasore has been used to probe the role of endocytosis in productive infection of HIV-1 in several different host cells. The inhibition on HIV-1 infection by dynasore has been interpreted as the involvement of dynamin-dependent endocytosis that leads to productive HIV-1 infection. Although dynasore was shown to be relatively specific and did not appear to affect dynamin-independent cellular functions [3], recent studies using dynamin triple knockout cells have revealed the robust off-target effects of dynasore on membrane ruffling and fluid-phase endocytosis [9]. These studies indicate that dynasore can inhibit dynamin-independent cellular functions and highlight the presence of unknown targets of dynasore that remain to be identified. It also raises the caution on the use of this inhibitor in probing dynamin-dependent cellular functions.

### Objective

The cellular route of HIV-1 entry for productive infection in CD_4+_ host cells is a fundamental question for the molecular understanding of HIV-1 infection and transmission [10] [11]. To elucidate the role of dynamin-dependent endocytosis on productive infection of HIV-1, we have thus evaluated the effect of dynasore on HIV-1 infection in several well-established cell lines.

## Results & Discussion

**Figure 1.**
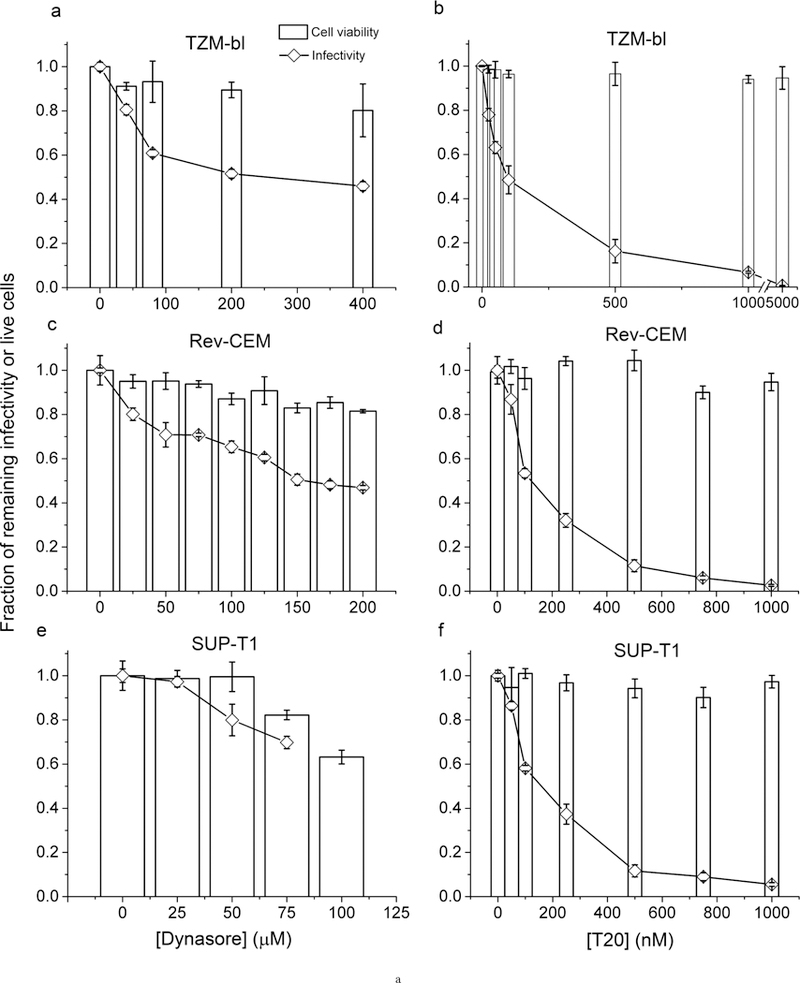

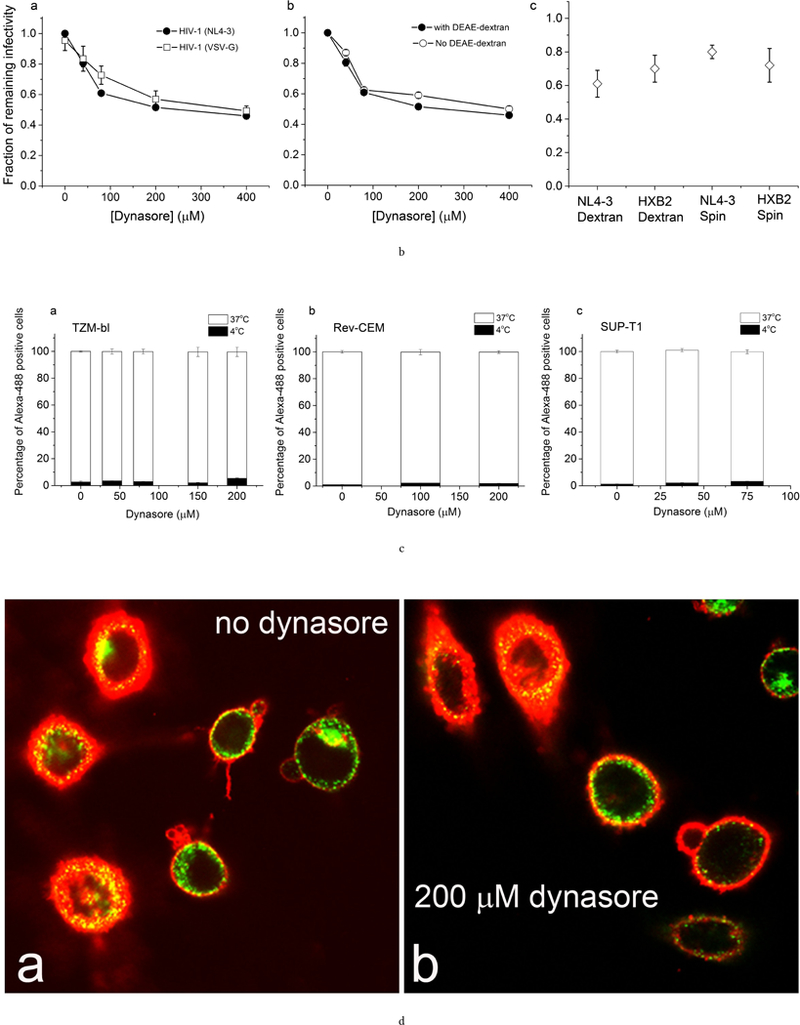
Effects of dynasore on HIV-1 infection and transferrin endocytosis in cell culture. **(A)** Inhibition of productive HIV-1 infection by dynasore or T20 in TZM-bl, Rev-CEM, and SUP-T1 cell lines. The fractions of remaining HIV-1 infectivity (diamonds) or fractions of live cells (white bars) upon dynasore treatment is shown in (a), (c) and (e) for TZM-bl, Rev-CEM and SUP-T1 cells, respectively. The fractions of remaining HIV-1 infectivity or fractions of live cells upon T20 treatment is shown in (b), (d) and (f) for TZM-bl, Rev-CEM and SUP-T1 cells, respectively. All the infectivity values were normalized to the infectivity measured in each cell line in the absence of dynasore or T20 treatment. Error bars represent standard deviations from 3 independent repeats of the same experiments. **(B)** Inhibition of productive HIV-1 infection in TZM-bl cells by dynasore under various conditions. (a) The fractions of remaining infectivity for HIV-1 NL_4–3_ virions (filled circles) or HIV-1 pseudotyped with VSV-G (hollow squares) upon treatment of the cells with various concentrations of dynasore. 20 μg/ml DEAE-dextran was included in the incubation mixture. (b) The fractions of remaining infectivity for HIV-1 NL_4–3_ virions in the absence of DEAE-dextran (hollow circles) or presence of 20 μg/ml DEAE-dextran (filled circles) upon treatment of the cells with various concentrations of dynasore. (c) The fractions of remaining infectivity for various HIV-1 virions in the presence of 20 μg/ml DEAE-dextran (Dextran), or in the absence of DEAE-dextran but under spinoculation (Spin). All cells were pretreated with 80 μM dynasore for 30 min at 37°C. NL_4–3_: NL_4–3_ virions with NL_4–3_ envelope glycoproteins; HXB2: NL_4–3_ virions pseudotyped with HXB2 envelope glycoproteins. Error bars represent standard deviations from 3 independent repeats of the same experiments. **(C)** Measurement of inhibition on transferrin uptake by dynasore in TZM-bl, Rev-CEM, and SUP-T1 cell lines. The percentages of Alexa-488 positive cells upon incubation with Aelxa-488 conjugated transferrin at 37°C for 5 min (white bars) or at 4°C throughout (black bars) is shown in (a), (b) and (c) for TZM-bl, Rev-CEM and SUP-T1 cells, respectively. All cells were pretreated with dynasore at various concentrations as indicated for 30 min at 37°C. All the percentages were normalized to the cell number measured in each cell line in the absence of dynasore treatment at 37°C. Error bars represent standard deviations from 2 independent repeats of the same experiments. **(D)**Confocal images of TZM-bl cells upon uptake of Alexa-488 conjugated transferrin in the absence of dynasore (a) or after treatment with 200 μM dynasore (b).

To investigate the entry of HIV-1 into host cells, we have chosen to work with three different cell lines that are well established in the literature for study of HIV-1 infection in cell culture: (1) TZM-bl cells, a HeLa-derived indicator cell line that was engineered to overexpress CD_4_ and CCR_5_ receptors, which is widely used for assaying the infectivity of HIV-1 virions in cell culture [12]; (2) Rev-CEM cells, an engineered indicator T cell line that has been demonstrated to report authentic HIV-1 infection [13] [14]; and (3) SUP-T1 cells, a non-engineered T cell line derived from an 8 year old boy who had a relapse of T-cell non-Hodgkin’s lymphoma [15]. To determine the effects of dynasore on HIV-1 infection in the above cell lines, we have treated these cells with various concentrations of dynasore for 30 min at 37°C before HIV-1 infection. These cells were then infected by HIV-1 for 2 hours at 37°C (Materials and Methods), followed by continued incubation at 37°C until the quantitation of HIV-1 infection. To control for the potential toxicity of dynasore on these cultured cells, we also prepared mock infection in the absence of virus but in the presence of various concentrations of dynasore, and measured the number of viable cells at each concentration by trypan blue staining. As shown in figure 1A (a, c and e), the fractions of remaining HIV-1 infectivity relative to those at zero concentration of dynasore are plotted in solid curves as a function of dynasore concentration in the culture ([Dynasore]) for these cell lines. The fractions of viable cells relative to those at zero concentration of dynasore are also plotted in white bars at each [Dynasore]. From these plots, it is clear that dynasore exerts inhibitions on productive HIV-1 infection at nontoxic concentrations, although the degree of inhibition varies from cell line to cell line. At 200 μM dynasore, both TZM-bl cells (Fig. 1Aa) and Rev-CEM cells (Fig. 1Ab) display inhibition of productive HIV-1 infection by 50%. However, this inhibition is subtle for SUP-T1 cells within the range of [dynasore] that we can test. Significant cell death occurs at 100 μM dynasore or above for SUP-T1 cells. For a side-by-side comparison with other inhibitors, we also carried out studies in the above cell lines using another well-established HIV-1 entry inhibitor, T20. The action of T20 is to bind the pre-hairpin intermediates of envelope glycoproteins during membrane fusion, which prevents six-helical bundle formation and thus blocks membrane fusion and HIV-1 infection [16]. As shown in figure 1A (b, d and f), the fractions of remaining HIV-1 infectivity relative to those at zero concentration of T20 are plotted in solid curves as a function of T20 concentration in the culture ([T20]) for these cell lines. The fractions of viable cells relative to those at zero concentration of T20 are also plotted in white bars at each [T20]. For all 3 cell lines, T20 can achieve close to 100% inhibition of HIV-1 infection under nontoxic concentrations. This comparison between dynasore and T20 also suggests that dynasore is not as potent as T20, and the pathways of HIV-1 infection blocked by dynasore may thus represent only a fraction of the pathways available for HIV-1 infection in these host cells.

The observed inhibition in TZM-bl cells by dynasore is qualitatively consistent with Miyauchi et al. [6], who first reported the inhibitory effect of dynasore on productive HIV-1 infection in TZM-bl cell line. Indeed, the same isolate of HIV-1 but pseudotyped with the envelope glycoprotein of vesicular stomatitis virus (VSV-G) produced quantitatively very similar patterns of inhibition in TZM-bl cells (open squares in Fig. 1Ba), suggesting that it is impairment of an endocytic process that partially blocks the infection of both viruses. Furthermore, the trend of inhibition remains quantitatively the same when we conducted infection in the absence of DEAE-dextran (Fig. 1Bb), suggesting that it is not the presence of DEAE-dextran that sensitized the virus to an endocytic process. Moreover, this inhibitory effect remained when we changed the viral envelope to that of HXB2, one of the prototype HIV-1 clones from infected human T cell lines [17] (Fig. 1Bc), although the degree of inhibition for HXB2 infection that we have observed at 80 μM dynasore was less than that by Miyauchi et al. [6]. Furthermore, for both NL_4–3_ and HXB2 enveloped viruses, spinoculation onto TZM-bl cells pretreated with dynasore produced comparable inhibition as infection in the presence of DEAE-dextran (Fig. 1Bc). Thus, this inhibition by dynasore on productive HIV-1 infection in TZM-bl cell line is real, regardless of the methods used to facilitate HIV-1 infection.

To probe the mechanism of dynasore inhibition of HIV-1 infection, we then assayed the transferrin uptake in the above 3 cell lines. Cellular uptake of transferrin has been characterized as a classical example of clathrin-dependent endocytosis, in which dynamin is required for clathrin-coated vesicles to pinch off from the plasma membrane [18]. Transferrin binds at the cell surface to its corresponding receptor and is internalized by receptor-mediated endocytosis, a process that requires the formation of clathrin-coated pit [19] [20] [21]. We incubated Alexa-488 labeled transferrin with various host cells that were pretreated with dynasore at 37°C for 30 min, and then used acid wash to remove surface-bound transferrin molecules before subjecting cells to flow cytometry for analysis. Across the range of dynasore concentrations that we have investigated for viral infection in each cell line, no apparent inhibition of transferrin uptake was observed at 37°C (white bars in Fig. 1C), the condition under which we conducted virus infection. In contrast, transferrin uptake can be almost completely blocked by shifting cells to 4°C for transferrin binding and uptake (black bars in Fig. 1C). These results indicate that transferrin uptake is fully functional in these cell lines despite the treatment with dynasore at concentrations that HIV-1 infection has been compromised.

As a further support to the above flow cytometry results, we have conducted confocal imaging for TZM-bl cells after incubation with Alexa-488 conjugated transferrin. As shown in figure 1D, Alexa-488 containing endosomal vesicles can be clearly observed both in the absence and presence of dynasore, indicated by the green punctate signals around the inner edge of the plasma membranes. Moreover, live cell imaging experiments using Alexa-594 conjugated transferrin revealed no apparent inhibition of transferrin uptake by dynasore at any time during the experiments. As shown in supplementary Movies 1 and 2, fluorescent endosomes were clearly visible both in the absence and presence of dynasore. The dynamic movements of these endosomes inside the cells are quite evident in both cases.

## Conclusions

In this study, the effect of dynasore on productive HIV-1 infection in 3 commonly used cell lines has been tested. Inhibition on HIV-1 infection was observed in all 3 cell lines, although the degree of inhibition varied. However, the observed inhibition is not correlated with any reduction in transferrin uptake. Instead, transferrin uptake remains fully functional in all these cell lines despite the pretreatment of these cells with dynasore at concentrations that partially block HIV-1 infection. These observations revealed that HIV-1 entry does not share the same pathway as transferrin uptake in these cell lines, and suggest that the inhibitory effect of dynasore on HIV-1 infection is not due to the impairment of clathrin-dependent endocytosis. Rather, dynasore displays an apparent complex effect in its use as an inhibitor for HIV-1 infection in these cell lines. Recent studies using dynamin triple knockout cells have revealed the inhibitory effects of dynasore on fluid-phase endocytosis and peripheral membrane ruffling, even though all 3 isoforms of dynamin are absent [9]. Therefore, the molecular targets of dynasore during HIV-1 infection in these cell lines remain to be identified.

### Limitations

Because the above studies were all conducted in cell culture, the relevance of these results for HIV-1 infection in vivo remains to be tested.

The inhibitory effect of dynasore observed for HIV-1 pseudotyped with VSV-G (Fig. 1Ba) suggests that an endocytic process was affected by dynasore. However, the complex effect of dynasore renders it difficult to precisely assess the role of endocytosis for productive infection of HIV-1 virions in these cell lines. Independent methods or more specific inhibitors are required in order to draw definitive conclusions. Among these, the K44A dominant-negative mutant of dynamin is likely to be a good candidate, which can be used for more specific inhibition of dynamin-dependent endocytosis. The role of dynamin-dependent endocytosis on HIV-1 infection can then be determined.

## Supplementary Material

Movie 1. Live cell movie taken at 20 min after addition of 5 μg/ml Alexa-594 conjugated transferrin to TZM-bl cells cultured on chambered coverglass at 37°C with 5% CO_2_ in complete media. A 600–frame fluorescence movie was taken with an exposure time of 100 ms for each frame at a frame rate of 1 Hz. It is evident that Alexa-594 conjugated transferrin has been uptaken by the cells through endosomes. It is apparent that these endosomes are dynamic inside the cell with endosomal trafficking being evident. The movie is played at 100 Hz.

Movie 2. Live cell movie taken at 20 min after addition of 5 μg/ml Alexa-594 conjugated transferrin to TZM-bl cells cultured on chambered coverglass. The cells were pretreated with 108.5 μM dynasore at 37°C with 5% CO_2_ in complete media for 30 min before the addition of transferrin. A 600–frame fluorescence movie was taken with an exposure time of 100 ms for each frame at a frame rate of 1 Hz. Despite the pretreatment of cells with dynasore, it is evident that Alexa-594 conjugated transferrin has been uptaken by the cells through endosomes. It is also apparent that these endosomes are dynamic inside the cell with endosomal trafficking being evident. The movie is played at 100 Hz.
